# Hormesis and Its Place in Nonmonotonic Dose–Response Relationships: Some Scientific Reality Checks

**DOI:** 10.1289/ehp.9619

**Published:** 2007-01-04

**Authors:** Paul Mushak

**Affiliations:** PB Associates, Durham, North Carolina, USA

**Keywords:** bidirectional dose response, biphasic dose response, hormesis, nonmonotonic dose response

## Abstract

**Objective:**

This analysis is a critical assessment of current hormesis literature. I discuss definitions, characterization, generalizability, mechanisms, absence of empirical data specific for hormesis hypothesis testing, and arguments that hormesis be the “default assumption” in risk assessment.

**Data sources:**

Hormesis, a biological phenomenon typically described as low-dose stimulation from substances producing higher-dose inhibition, has recently garnered interest in several quarters. The principal sources of published materials for this analysis are the writings of certain proponents of hormesis. Surprisingly few systematic critiques of current hormesis literature exist. Limits to the phenomenon’s appropriate role in risk assessment and health policy have been published.

**Data synthesis:**

Serious gaps in scientific understanding remain: a stable definition; generalizability, especially for humans; a clear mechanistic basis; limitations in the presence of multiple toxic end points, target organs, and mechanisms. Absence of both arms-length, consensus-driven, scientific evaluations and empirical data from studies specifically designed for hormesis testing have limited its acceptance.

**Conclusions:**

Definition, characterization, occurrence, and mechanistic rationale for hormesis will remain speculative, absent rigorous studies done specifically for hormesis testing. Any role for hormesis in current risk assessment and regulatory policies for toxics remains to be determined.

The last decade has witnessed a revival of interest by some in hormesis, a biological phenomenon broadly defined as a “stimulatory” response to low doses of a substance that otherwise causes inhibition of response at higher doses (e.g., Calabrese and Baldwin 2002a).

Hormesis is a type of dose–response relationship, of which two general forms exist. Monotonic dose–response (MDR) relationships describe responses that proceed unidirectionally from zero dose or doses above zero. Nonmonotonic dose–response (NMDR) relationships show biphasic or bidirectional responses to dose, appearing in U-shaped or inverse U-shaped graphic forms. Graphic depictions of these responses appear in a number of articles (e.g., Calabrese and Baldwin 2001a, 2001b, 2001c; Davis and Svendsgaard 1994).

Hormesis in its current form has had surprisingly little systematic scientific scrutiny or collective peer review. Thayer et al. (2005) evaluated the relative merits and limits of hormesis as the “default assumption” for human health risk methodologies and associated policies. They identified lack of a mechanistic footing, questionable interpretations of hormetic data, and underestimation by proponents of the complexity and diversity of human population response to xenobiotic exposures.

The specific scientific framing questions for hormesis in this analysis are as follows:

- What is a coherent and valid working definition for hormesis?
- What is its generalizability?
- What is the evidence for or against hormetic phenomena in humans?
- Has any general underlying mechanism been adequately documented?
- What are limits to borrowed mechanisms?
- Can typical single end point hormesis accommodate multiple toxic end points, target organs, and toxicologic mechanisms?
- Is hormesis a valid default assumption in human risk assessment and exposure reduction policies?

## Defining Hormesis

Hormesis has been plagued by absence of a stable definition. First, development of a definition has been an ad hoc process, with backing and filling over time. This ad hoc process has not been shaped by hypothesis testing but by the persistent search for, and analysis of, existing bodies of data [see Calabrese and Baldwin (2002a) for a current definition and its changes over time].

Definitions of hormesis also arose with little disciplinary consensus in relevant fields from expert committees using objective, arms-length approaches and guidelines.

Calabrese and Baldwin (2002a) define hormesis comprehensively as

an adaptive response characterized by biphasic dose responses of generally similar quantitative features with respect to amplitude and range of the stimulatory response that are either directly induced (i.e., direct stimulation hormesis [DSH]) or the result of compensatory biological processes following an initial disruption in homeostasis (i.e., overcompensation stimulation hormesis [OCSH]).

There are still differences of opinion on this definition. It is largely driven by speculation and difficult to test empirically at present (Kitchin 2002; Pickrell and Oehme 2002). Others question the appropriateness of the term “hormesis” to cover all low-dose responses (Chapman 2002). “Direct” stimulation, one-half the definition, is descriptive only and lacks a mechanism. Overcompensation stimulation linked to preservation of homeostasis is offered as the mechanism of hormetic action.

Proponents of hormesis often attach the notion of “benefit” to hormesis. If an adverse effect has the dose–response curve going one way, then the opposite direction is deemed beneficial. This is argument by anomaly rather than by empirical evidence. The Calabrese and Baldwin (2002a) definition uncouples “benefit” from hormesis, given that hormesis is not inherently “beneficial” and can be deleterious to health. Anticancer drug actions that inhibit tumorigenesis in the normal dose–response range may be preceded by tumor cell proliferation, that is, an adverse end point, at low doses (Calabrese 2005a). Some still believe that hormetic responses are typically beneficial (Calabrese 2005b; Eaton and Klaassen 2003).

## What Is Its Generalizability?

Various articles by Calabrese and Calabrese and Baldwin [see Calabrese (2005b) for citations] traced the early history of what is known as hormesis and claim it was “marginalized” unfairly. These authors assert that early 20th century research established hormesis sufficiently to warrant scientific acceptance but for unfavorable biases from influential scientists and linkage to homeopathy. Other claimed impediments are the evolution of risk assessment models favoring the high-dose realm of dose responses and public pressures for dealing with high-dose not low-dose exposures.

Review of the cited studies yields little convincing evidence that a strong earlier bias existed. More prosaic reasons likely applied. First, it would be reasonable to expect that experimental anomalies would garner less attention than the research’s major focus. Second, such anomalies would not be easily interpretable, given the stage of science at the time. Third, risk assessment itself and risk assessment models using dose–response data are of recent vintage and chronologically unconnected with any early scientific views of hormesis. Risk assessment approaches largely trace to the 1983 seminal monograph *Risk Assessment in the Federal Government: Managing the Process* by the National Academy of Sciences/National Research Council (NAS/NRC 1983). The beginnings of U.S. regulatory agency uses of risk assessment are traced there mainly to the late 1970s. The U.S. Environmental Protection Agency (EPA) computerized database of dose–response information, the Integrated Risk Information System (IRIS; U.S. EPA 2007), did not see routine online use until the early 1990s.

The current period of published interest in hormesis dates to about 1992, but a still problematic definition did not appear until 10 years later (Calabrese and Baldwin 2002a). This lag helps quell arguments that slow or little acceptance of earlier incarnations of hormesis was scientific hostility and not simply the workings of science.

The current reception accorded hormesis by the scientific and health communities has been mixed. Cook and Calabrese (2006) overstate acceptance and understate wide skepticism. Considerable skepticism about the nature and scope of hormesis in humans exists (see below) and is science based. Characterization and application of hormesis sufficient for scientific acceptance are hobbled by flaws in existing data and the paucity of statistically and empirically well-designed studies for hormesis hypothesis testing.

Definition of a phenomenon is not the same as its identification and characterization. Attempted identification and characterization of hormesis are occurring simultaneously with developing a definition. Investigators must develop criteria for recognition, characterization, and generalizability. They especially must establish that it is biologically and toxicologically significant, and not merely a gross biodynamic or biokinetic artifact that is relatively invariant in magnitude or range across systems and substances.

It is clear something is occurring at low dose in some dose–response relationships anomalous to the rest of the curve (e.g., Calabrese and Baldwin 2001a, 2001c, 2002a). Criteria for presence or absence of hormetic responses in published studies, like its other elements, lack consensus from the scientific community. Two critical questions are whether both “numerator” and “denominator” for establishing hormesis frequency in nature can be reliably captured by published literature and whether examined literature captures published literature.

What is the minimal frequency for the occurrence to be termed “generalizable?” Proponents use ambiguous measures for generalizability, including “widely and independently observed” (Calabrese and Baldwin 2001c), “frequently encountered” (Calabrese and Baldwin 2001b), and “ubiquitous” (Cook and Calabrese 2006). These terms for hormesis in its current state conflict with usual dictionary definitions for “generalizability.” For example, “ubiquitous” means “existing or being everywhere at the same time” (Merriam-Webster Inc. 2002).

Davis and Svendsgaard (1994) examined a preselected database to determine the frequency of hormetic responses sufficient to clearly show U-shaped or inverse U-shaped curves. They used peer-reviewed literature in the U.S. EPA’s IRIS database for toxicants, focusing on information for determining a reference dose (RfD). The RfD is the daily lifetime intake of a substance not likely to be harmful. They found that about one-sixth (17%) of toxicology articles met their selection criteria, the fraction giving some indication of nonmonotonic relationships was 22%, and 93 or 12% of 780 showed an adequate U-shape or inverse U-shape dose–response relationship. They concluded that such responses are roughly 12–24% of published dose responses using their methodology.

Calabrese and co-workers also evaluated the generalizability of the hormetic response in published literature (Calabrese and Baldwin 1997, 2001a; Calabrese and Blain 2005; Calabrese et al. 1999). Their gathered database provided about 20,000 articles from selected journals using ad hoc criteria (Calabrese and Baldwin 1997). A point system was developed (Calabrese and Baldwin 2001a; Calabrese et al. 1999), with more NOAEL (no observed adverse effect level) doses getting more points. This approach was criticized by Crump (2001) on statistical grounds. For example, it is important to know the extent of statistical error and level of significance for NMDR relationships in sampled literature. The next stage used combined selection criteria for ad hoc entry and ad hoc evaluation. This methodology produced 37% of studies meeting evaluation criteria and, of the 1,089 doses below the NOAEL, 213 (19.5%) satisfied statistical criteria (Calabrese and Baldwin 2001b).

How well do the various screenings by Davis and Svendsgaard (1994) or by Calabrese and co-workers (Calabrese and Baldwin 1997, 2001b; Calabrese et al. 1999) reflect hormetic generalizability? At this time, we do not know, as the approaches remain unvalidated. Any ad hoc screening process for a particular purpose requires validation for reliable analysis and interpretation. One problem with the approach of Calabrese and co-workers (Calabrese and Baldwin 2001b; Calabrese et al. 1999) is dependence of frequency estimation on entry criteria. Calabrese and co-workers (Calabrese and Baldwin 2001b; Calabrese et al. 1999) require at least two doses below the NOAEL. One can use an equally or more reasonable criterion of three or four doses, decreasing the frequency.

Calabrese and Calabrese and Baldwin reported illustrative possible, likely, or observed bidirectional responses in various endogenous and exogenous classes of substances or biological processes. Calabrese claimed hormesis was present in studies of estrogen and related compounds (2001f), nitric oxide (2001h), prostaglandins (2001j), chemotherapeutics (2005a), opiates (2001i), adenosine (2001a), dopamine (2001e), serotonin (2001g) androgens (2001b), apoptosis (2001c), and cell migration/chemotaxis (2001d). Calabrese and Baldwin reported hormesis for chemotherapeutics (2003a) and such xenobiotic substances as ethanol (2003b), peptides (2003e), diverse toxics studied in the National Toxicology Program for dose range (2003c), and metals (2003d). The large survey of metals (Calabrese and Baldwin 2003d) largely covers simple experimental systems with few human data.

Surveys of Calabrese and co-workers, as cited in the previous paragraph, do not quantify the global frequency of hormesis within specific classes of substances. They merely report instances of hormesis-type responses across groups. For example, lead entails several dozen or fewer citations over decades and for limited experimental systems, a tally dwarfed by the total number of lead citations, even when taking account of study designs. In 1991 alone there were about 1,200 abstracted articles listed in *Chemical Abstracts* (American Chemical Society 1991) for human, experimental and other laboratory studies, 600 abstracted articles on lead in experimental systems in *Biological Abstracts* (Union of American Biological Societies 1991) and about 400 articles for both human and test animal exposures in *Index Medicus* (National Library of Medicine 1991; Mushak 1992).

## What Is the Evidence for or against Hormetic Phenomena in Humans?

A critical question for hormesis is its presence and characterization in humans. A convincing database in epidemiology, whatever the simple experimental evaluations, is required to elevate hormesis from scientific curiosity to something providing possible ground truth for science and health policy. A principal concern with hormesis in humans is relevance to exposures to large numbers of chemical contaminants, many being toxic at higher doses. Second, human populations by their nature are diverse genetically and physiologically, making their study for hormesis difficult, especially for individual members highly sensitive to toxicologic or other biological challenge. Proponents of hormesis readily point to moderate ethanol consumption as producing beneficial hormetic behavior in humans (Calabrese and Baldwin 2003b). However, fetal ethanol exposures during moderate maternal consumption during pregnancy can produce neurotoxicologic fetal injury such as abnormal neuronal migration and abnormal development of the dendritic spines, potentially producing mental retardation (Abel et al. 1983).

### Radiologic hormesis

Some proponents of radiologic hormesis hold that the linear no-threshold (LNT) dose–response relationship for radiologic carcinogenesis in humans and experimental animals is no longer tenable and radiologic hormesis should be the prevailing model (e.g., Calabrese 2005b; Pollycove and Feinendegen 2001). However, research into radiologic carcinogenesis in humans continues to feed a huge epidemiologic database documenting the persistence of radiologic carcinogenicity with lower dose. Little convincing evidence exists to support human radiologic hormetic responses nullifying the LNT model of low-dose carcinogenesis. Current thinking also is mixed about a conceptual context for human radiologic hormesis (Johansson 2003; Pollycove and Feinendegen 2001; Upton 2001).

Recent expert consensus treatises on low-dose carcinogenicity of ionizing radiation in humans present analyses largely supporting the LNT model. The most recent report by the NAS/NRC on the biological effects of ionizing radiation, *BEIR VII-Phase 2* (NAS/NRC 2006), endorsed preservation of the LNT model, that is, cancer risk proceeds in a linear fashion at lower doses without a threshold. Appendix D of the report covers radiation hormesis. The report concluded that animal and cell studies suggesting benefits or threshold to harm from ionizing radiation are “not compelling” and that, at present, any assumption of net health benefits of radiation hormesis over detrimental impacts at the same dose is unwarranted.

The December 2004 draft report of the International Commission on Radiation Protection (ICRP 2004), the report of the United Nations Scientific Committee on the Effects of Atomic Radiation (2000), and the report of the National Council on Radiation Protection and Measurements (NCRP 2001) have all concluded the LNT model remains valid. The 2005 report of the joint French National Academy of Medicine and the Institute of France Academy of Sciences on low dose-carcinogenic effect relationships for ionizing radiation (Académie Nationale de Médecine 2005) raises doubts about use of, or recommendations for, the LNT model at low (< 100 mSv) and very low (< 10 mSv) doses. The French report refers to radiation hormesis only in passing and in narrow context.

Several recent epidemiologic studies are consistent with an LNT dose response. The Techa River study in the Southern Urals region of Russia (Krestinina et al. 2005) reported individualized risk estimates for excess cancers from radioactivity exposures traced to a weapons plant leak in the 1950s. The extended cohort, with 29,873 people born between 1950 and 1960, provided strong evidence of low-dose radiologic carcinogenesis. Cardis et al. (2005), in their studies of 400,000 nuclear plant workers in 15 countries, concluded that 1–2% of cancer deaths among the cohort may be due to radiation. The results for the nuclear workers produced conclusions similar to those of the Techa River findings and both articles support the ICRP standard of 20 mSv/year for occupational protection.

### Chemical hormesis

I do not deny that claimed chemical “hormesis” may exist in some experimental data sets, whatever its nature and overall frequency. However, clearly demonstrable and interpretable hormetic responses are not readily available from human data. Rather, human data lend overall support to conventional LNT or threshold dose–response relationships. Two examples are consistent with LNT dose response in humans, low-level lead neurotoxicity in children and low-dose arsenic cancer risks.

Numerous reports document lead’s developmental neurotoxicity and other developmental effects in children without threshold [e.g., Centers for Disease Control and Prevention (CDC) 2005; Lanphear et al. 2005; NAS/NRC 1993]. Developmental neurotoxic effects detected in well-designed longitudinal studies extend to blood lead (PbB) levels well below 10 μg/dL (range, 3–7 μg/dL) for children (Lanphear et al. 2005). The authors’ pooled analysis of internationally gathered longitudinal epidemiologic data actually shows the neurotoxic response relationship at PbBs < 10 μg/dL as more robust than for values > 10 μg/dL.

Inorganic arsenic (iAs) carcinogenicity continues to be documented in large epidemiologic studies at lower and lower doses. Data sets for low-dose iAs carcinogenicity include those from Taiwan (e.g., Chen et al. 1992; Chiou et al. 2001; NAS/NRC 1999, 2001) and from South American studies (e.g., Ferreccio et al. 2000; Smith et al. 1998, 2006). Other data appeared from Finland (Kurttio et al. 1999), China, India, and Bangladesh [e.g., NAS/NRC (2001) and references therein].

The NAS/NRC 1999 report concluded there is no compelling reason for use of a cancer risk model other than the LNT approach. In fact, exposures producing measurable iAs cancer rates approach earlier low-dose extrapolation rates. Chiou et al. (2001) found increased relative risks of 1.6 and 1.9 versus controls for urinary tract and transitional cell cancers respectively with water As intake of 10–50 ppb.

### Hormesis and human risk populations

Little evidence exists that hormetic phenomena play a role in either defining human populations at elevated toxicity risk or in ameliorating or otherwise affecting heightened risk, particularly with prenatal or early postnatal exposures. Calabrese and Baldwin (2002b) showed there is little evidence in the hormesis literature linking hormesis to differential sensitivity of human or other populations to various toxic effects. Cook and Calabrese (2006) erroneously used the Calabrese and Baldwin (2002b) article to argue there is a hormetic role in toxicologic sensitivities, but the earlier article does not identify one.

Recent articles on hormesis largely fail to address hormesis as a modifier of environmental xenobiotic impacts on embryologic and/or fetal development in humans or the linkage of prenatal toxic exposures to postnatal developmental effects. There is a particularly high risk for genetic or developmental toxicologic harm in humans and experimental animal systems exposed *in utero*. Noted above was the ethanol example in pregnancy in rebuttal to Calabrese and Baldwin (2003b). Any putative role for hormesis is further blurred by developments in two areas. First, there is the ability of potent human carcinogens such as iAs and certain polycyclic aromatic hydrocarbons (PAHs) to function as transplacental carcinogens in humans or animals. Second, there are endocrinologic toxic responses, particularly those imparted *in utero*, induced by endocrine disruptors. Drinking water iAs appears to be a transplacental carcinogen in humans and experimental systems. Prenatal exposures produce postnatal carcinogenic effects. Here, carcinogenesis latency is also a developmental latency. Arsenic crosses the human placental barrier (Concha et al. 1998), whereas extensive epidemiologic data associate arsenic exposures with lung and hepatocellular carcinomas (NAS/NRC 1999, 2001).

Data from a Chilean population exposed to drinking water iAs show clear increased mortality from lung cancer and bronchiectasis in younger adults when exposures occurred *in utero* and early childhood (Smith et al. 2006). Individuals born just before the peak exposure period and exposed during early childhood and those born during the high exposure period with *in utero* and early childhood exposure all displayed significantly increased standardized mortality ratios (SMRs) for lung cancer and bronchiectasis compared with the rest of Chileans 30–49 years of age. Experimental findings in two different strains of mice support this, showing iAs to be a complete transplacental carcinogen in mice, *in utero* exposures producing, for example, hepatocellular carcinomas in the mice as adults (Waalkes et al. 2003, 2004). Liu et al. (2006) found the mouse toxicogenomic changes *in utero* with iAs exposure to include oncogene expression stimulation, liver feminization, cell cycle dysregulation, and disruption of cell–cell communication.

Certain PAHs appear to act as transplacental developmental neurobehavioral toxicants and carcinogens. Perera et al. (2006) reported that highest quartile prenatal PAH exposures in humans were linked to lower Bayley scores at 3 years of age, whereas Yu et al. (2006) showed that exposures of pregnant mice to dibenzo[*a,l*]pyrene produced both increased mortalities in offspring at 12 weeks from an aggressive T-cell lymphoblastic lymphoma and lung tumors in all animals surviving to 10 months, with some having liver tumors.

Endocrine disruptors have extremely high toxicologic potency at extremely low doses, especially *in utero* (e.g., Melnick et al. 2002; Sheehan et al. 1999; Welshons et al. 2003). How does hormesis deal with endocrine disruptors? The literature for endocrine disruptors indicates that they may act without threshold and even the LNT approach understates risk at extremely low doses. Disruptor action involves saturation kinetics at receptor sites, where early saturation and steep dose responses are underestimated as to adverse disruptor effects by even low-dose linear extrapolations (Sheehan 2006; Sheehan et al. 1999; Welshons et al. 2003).

Sheehan et al. (1999) found that there did not appear to be a threshold dose for estradiol-induced sex reversal of turtle embryos, the lowest dose tested (40 pg/g, ppt) showing 14% sex reversal in the turtle embryos. Sheehan (2006) also analyzed 31 additional data sets. Use of a normalized Michaelis-Menten equation for data fitting showed good fit from about 1 to > 95% receptor occupancy and contradicted the threshold assumption. Welshons et al. (2003) noted that exogenous estrogens participate in a physiologic system that is active and already above any threshold due to endogenous estrogens. They also noted linear low-dose extrapolation, a typical risk assessment operation with substances such as carcinogens, may be inappropriate and results in underestimated risk above the physiologic zone where estrogen activity is receptor mediated. Deviation of the linearization line from the receptor occupancy curve is evident in their Table 2.

Finally, hormesis discussions generally ignore human genetic susceptibilities as a factor in risk. Human toxicology data clearly show the role of genetic variability in biotransformation of xenobiotics and one can consult any works on pharmacogenetics for details. As one example, genetic variability for iAs biotransformation (biomethylation) as a potential factor in its carcinogenic potency has been documented (Chung et al. 2002; Vahter 2000; Yu et al. 2003).

## Attempted Characterization of the Hormetic Phenomenon’s Underlying Mechanisms

Little in the way of actual *de novo* experimentation to specifically characterize the mechanistic or biokinetic nature of hormesis has been carried out. Mechanistic interpretations are often speculative and descriptive, not data driven.

Calabrese and Baldwin (2002a) stated that the mechanistic basis of the phenomenon is an overcompensation induced by response to disturbance of homeostasis. Any role for direct stimulation has not been determined. Besides a general mechanism of compensation, various mechanisms were borrowed from literature describing behaviors of certain substances.

Adaptive, compensatory (overcompensatory) mechanisms in response to disturbed homeostasis first require there be a time lag during which the disturbance in homeostasis occurs (Calabrese and Baldwin 2001c; Rozman and Doull 2000). The time dependency is expressed in a prehormetic low-dose region of the dose–response curve for some end point that is monotonic early in time, that is, is in the opposite direction of the later hormetic or “compensatory” segment of that curve (e.g., Calabrese and Baldwin 2001c). Does significant toxicity precede hormesis? Disturbances in claimed homeostasis in any time period preceding onset of hormesis cannot be considered pretoxicologic or toxicologically irrelevant. No empiric or conceptual evidence for this exists.

If hormesis-linked homeostasis is a highly evolved, efficient process for organismal preservation, it should logically respond sparingly if at all to trivial, nontoxic challenges. Acceptance of homeostasis preservation as the global mechanism for hormetic phenomena equally requires acceptance of the occurrence of some toxicologic event triggering hormesis. Second, there is little evidence that any and all low-dose toxicologic effects occurring prehormesis (within the curve’s hormetic zone) would be transitory over time and abate with onset of hormesis, whatever the time-dependent curve shape. There is evidence supporting the opposite. Developmental neurotoxic metals produce low-dose effects that are essentially irreversible.

The observation of modest, relatively invariant “hormetic” responses across various end points and various classes of substances for dose–response curves with a hormetic zone is quite puzzling. Homeostatic preservation would arguably predict compensatory (over-compensatory) responses proportional to magnitude of stressing events. However, the hormetic response over “controls” is 30–60% across many classes of substances and the range of response is within one order of magnitude or so. Why would highly efficient preservation of somatic homeostasis exert as much metabolic effort for responses to a low-toxicity substance as for responses to a high-toxicity substance?

Conolly and Lutz (2004) have advanced an alternative approach to hormetic mechanisms. Nonmonotonicity seen in crude graphic depictions of hormetic events is postulated to be multiple effects with monotonic dose–response curves superimposed on each other, involving, for example, membrane receptors with subtypes of varying ligand affinities, androgen receptor-mediated gene expression, repair of background DNA damage by enzymatic activity induced by adducts, and rates of mutation as a consequence of DNA damage multiplied by rate of cell division. Hormetic zones are rationalized as a net expression of discrete and concurrent monotonic molecular events. The Conolly and Lutz scheme, however, does not appear to explain the modest or invariant nature of hormetic responses. Similarly, Kitchin and Drane (2005) noted that multiple and different mechanisms may attach to “beneficial” versus “toxic” effects, with aggregated impacts in either direction contradicting any notion of a single mechanism explanation.

## What Are Limits to Borrowed Mechanisms?

It has not been established nor is it logical that mechanisms proposed for claimed forms of hormesis for different classes of substances—drugs, essential nutrients, endogenous bioactive substances—can be legitimately borrowed to explain what is occurring mechanistically with xenobiotics. Proponents of hormesis have nevertheless borrowed liberally from their literature. Mechanisms of action of classes of drugs are predetermined by purpose of the drug, the systemic target(s) of the drug, and some pharmacodynamic idea of therapeutic limits, and not by treating the pharmacologic characterization of the drug as an antitoxicologic mechanism. Mechanistic rationales for hormesis also borrow from such areas as biotransformations of xenobiotics, substance interactions deemed potentially protective, and actions of essential nutrients.

The ability of xenobiotic organic compounds to induce enzyme systems for their metabolic biotransformation and detoxification is not fully protective or compensatory to the organism. Oxidative biotransformations of PAHs produce readily excretable hydrophilic conjugation products and protective detoxification. They can produce increased adverse effect. The potent PAH carcinogen benzo[*a*]pyrene (BaP) is metabolized to a stereoisomeric mixture of diol epoxides. One stereoisomeric form is held to be the proximate carcinogen (e.g., Parkinson 2001). Such biotransformations typically occur with a latency period and at relatively high dosing, rendering any likely “protective” or “beneficial” role with low, sensitive toxic end points problematic.

Biomethylation of iAs has been claimed to be both a detoxification mechanism and one more complete at low doses (e.g., Buchet and Lauwerys 1994). More recent studies support biomethylation as a step in the substance’s carcinogenic action (e.g., Mass et al. 2001). In the area of many known multielement interactions, the zinc (Zn)–cadmium (Cd) interaction may appear hormetic, inducing biosynthesis of metallothionein (MT) and binding of Cd to yield the Cd–MT complex. Although this complex is held to reduce Cd binding and toxicity using certain toxicologic end points, it can produce early nephrotoxicity in the form of proximal tubular dysfunction [International Programme on Chemical Safety (1992) and references therein].

The present placement of nutrients and nutrient actions within conventional dose–response frameworks from essentiality to toxicity allows some to define nutrient essentiality as an example of hormesis. There are obvious problems with this linkage.

Nutrients are biochemically and physiologically unlike xenobiotics or other exogenous substances in not expressing toxic effects as an intrinsic behavior central to their nutritive role. Nutrients typically express such a high therapeutic index (ratio of some defined toxic dose to some defined therapeutic dose) that dual bioactivity is more accurately captured by at least two separate dose–response curves. Neither of these relationships convincingly expresses a readily defined hormetic response. Nutrient “dose–response” relationships mainly cover intakes producing nutrient deficiencies, nutrient optimal activity, and preservation of nutrient safety with excess intakes rather than global, combined dose-responses that define nutrient essentiality as a small early portion of a mainly dose–toxicity curve. See Figures 2–4 to 2–6 of the World Health Organization text (WHO 1996) for graphic depictions of minimum, normative, and maximum population zinc intakes defining the “dose” portions of zinc’s nutritive dose responses.

Nutrients partake of a homeostasis through control of uptake, distribution, retention, deposition, or excretion (WHO 1996). Nutrient homeostasis should not be considered equivalent to any homeostatic mechanism for xenobiotic hormesis. Control of nutrient biokinetics and essentiality works more to prevent physiologically harmful nutrient deficiencies and sustain optimal nutrient utilization than it does to prevent toxic effects of excessive nutrient intakes.

## How Can Single End Point Hormesis Accommodate Multiple Toxic End Points, Target Organs, and Toxicologic Mechanisms?

The current hormesis literature is largely based on isolated, single end points across a range of doses. However, toxicants often operate to produce multisite toxicity via multiple end points and likely multiple mechanisms, meaning data are needed to determine which end point is the most toxicologically sensitive. This end point should then determine the dose–response curve and hormetic phenomena therein.

There is also the matter of multiple effects and their mechanisms producing erroneous conclusions that hormesis is actually present (Conolly and Lutz 2004; Kitchin and Drane 2005; Roberts 2001). Roberts (2001) made general reference to low-dose multiple mechanisms working in opposite directions creating erroneous interpretations. Conolly and Lutz (2004) provided concrete examples of the potential for misinterpretation when multiple low-dose processes are superimposed on each other in the intact organism. An empirical example is published data for Cd-induced sex organ tumorigenesis in rats (Goyer et al. 2004; Waalkes et al. 1988, 1997). The dose–response curve for prostatic tumors in rats dosed with Cd appears nonmonotonic at higher doses. This response is not “hormetic” but expresses tumorigenic interactions between prostatic and testicular injury. Prostatic tumorigenic effects in rats dosed with Cd occur at lower doses than testicular tumors, whereas testicular injury at higher doses occurs with altered androgen release. Altered release changes the tumorigenesis dose–response curve for prostate tumor rates at those higher doses.

A statistical dimension to multiple end point/multiple site relationships complicates attributions to hormesis. Calabrese (2002) has argued that organ-specific tumor induction rates at three levels of dosings with 2,3,7,8-tetrachlorodibenzo-*p*-dioxin in rats (Kociba et al. 1978) is beneficial, in that aggregate tumor rate is below the control rate. This is misleading. Thayer et al. (2005) correctly noted no reversal in tumor rates (curve direction) in any organ site for the doses used, with only increases in tumors being recorded in some sites versus controls. One cannot statistically dilute the data across tumor sites that show higher rates over controls, i.e., are more tumorigenically sensitive to dioxin exposures. One also cannot dismiss elevated risk of tumorigenesis for sensitive organ sites as the driver for overall dioxin risk assessment.

## Does the Available Evidence Support the Use of Hormesis as a Default Assumption in Human Risk Assessment and Exposure Regulation Policies?

The available evidence does not support the use of hormesis for evaluating low-exposure human health risk assessments and any implementing of regulatory and health policy, especially for xenobiotic, potentially toxic contaminants. Classes of drugs, essential nutrients, or endogenous bioactive substances are not the principal issue. The U.S. Food and Drug Administration regulates drugs and the NRC provides timely advisories on recommended daily allowances for nutritional components. We currently know little about the temporal, mechanistic, genetic, or risk susceptibility aspects of hormesis for use in any human or ecologic risk assessment default capacity. Using a default assumption requires the assumption be a reliable and quantitatively definable surrogate for specific but unavailable data. Hormesis fails in this regard.

Reasons for failure of hormesis in this capacity are multiple. Frequency of hormesis in nature or the literature shows it’s not universally present, being on the order of 20–25% or less of examined examples (Calabrese and Baldwin 2001b; Calabrese et al. 1999; Davis and Svendsgaard 1994). This frequency in turn cannot be ranked with other risk model frequencies as some suggest (Calabrese and Baldwin 2003f), since an independent, statistically acceptable global statistical sampling of all published literature for all dose–response models has not been done. The above frequencies are for experimental systems, with little convincing evidence of hormesis in humans.

Mechanistically, hormesis is a hypothesis still in the realm of speculative assessment, ruling out elevation to default assumption (Conolly and Lutz 2004; Kitchin and Drane 2005; Thayer et al. 2005). It is not the case that xenobiotics with a claimed hormetic zone in their dose–response relationships show only beneficial behaviors or even show more beneficial than adverse effects at low dose (see above). Proponents have uncoupled the idea of benefit or harm from their last effort at a definition (Calabrese and Baldwin 2002a). This therefore requires previous data before use. This analysis and the work of others show that hormesis does not accommodate human risk variability, whatever the nature of that variability. Cook and Calabrese (2006) erroneously assert hormesis does accommodate risk variability.

Hormesis does not accommodate multiple toxic end points, target organs or mechanisms. Use of single end points common in hormesis would understate or misrepresent the full toxicologic picture of low-dose effects for reasons detailed earlier. Proponents argue (Cook and Calabrese 2006) that the potential for hormesis to be a beneficial effect would promote human health. But how does one use hormesis without knowing in advance whether responses labeled hormetic are deleterious or beneficial? And how would one know this in advance without first exposing human populations?

Is hormesis as default superior to other risk models across the board? Class A chemical carcinogens and endocrine disruptors, two classes of xenobiotics that figure or may figure in many nonoccupational population exposure scenarios, have not figured prominently in attempted extension of hormesis to human risk assessment. Second, available epidemiologic data offer little argument in favor of hormesis as a preferred human risk model. Hormesis proponents leave unsaid how the factor of uncertainty is dealt with, in contrast to the threshold risk models that employ uncertainty factors. In fact, proponents have not yet laid out a convincing methodologic schematic that actually walks the reader or risk assessor through a hormesis-based quantitative risk assessment (Calabrese and Cook 2005).

## Conclusions

This review examined the phenomenon of hormesis as currently defined and characterized. It was presented within a seven-topic framework: definition, characterization, generalizability, relevance to humans, mechanisms, relevance to multiple toxic phenomena, and role in risk assessment and regulatory or public health policies.

One concludes:

- Definition, characterization, and generalizability of hormesis remain largely problematic.
- General mechanisms proposed for hormesis appear driven more by speculation than by empirical evidence from specific mechanistic testings, while similarities to other phenomena do not permit mechanistic borrowing.
- It is unlikely that single end point hormesis would be quantitatively useful for toxics expressing multiple toxic effects in multiple organs and through multiple mechanisms.
- Until hormesis is rigorously studied through new and specifically designed protocols for hypothesis testing, its nature remains speculative.
- Persisting, serious limits to all the above aspects of hormesis hobble and likely will continue to hobble its use for regulatory and public health policies.
